# Symmetry of Wild Boar Damage to Agricultural Crops: Results of over 20 Years of Damage Monitoring in Central Europe

**DOI:** 10.3390/ani15111587

**Published:** 2025-05-29

**Authors:** Paweł Nasiadka, Daniel Klich, Wanda Olech, Maria Sobczuk

**Affiliations:** Department of Animal Genetics and Conservation, Institute of Animal Sciences, Warsaw University of Life Sciences, Ciszewskiego 8, 02-786 Warsaw, Poland; daniel_klich@sggw.edu.pl (D.K.); wanda_olech@sggw.edu.pl (W.O.); maria_sobczuk@sggw.edu.pl (M.S.)

**Keywords:** agriculture, crop protection, conflict mitigation, wildlife management, wild boar

## Abstract

This study analyzed 9871 cases of wild boar damage recorded over more than 20 years using a consistent methodology in a 5000-hectare area with minimal changes in crop structure. This allowed for a reliable assessment of seasonal damage dynamics. Wild boar exhibited seasonal feeding preferences: grasses in spring, cereals in summer, legumes in early autumn, and root crops in late autumn. A bimodal pattern of damage was identified, comprising three types. The *rare and severe* pattern occurred in spring when the wild boar population at its lowest size caused infrequent but highly damaging events, mainly in meadows. The *frequent and weak* pattern appeared in late summer and autumn when wild boar numbers were two to three times higher, leading to frequent but less severe damage. The *indirect* pattern was linked to lower field attractiveness in summer and winter. The high symmetry of damage observed suggests that an effective wild boar management strategy should account for not only damage reduction but also sanitary risks, such as ASF.

## Simple Summary

This study looked at over 9800 cases of wild boar damage to crops, recorded consistently over more than 20 years in a farming area in central Europe. Because the area and the types of crops stayed almost the same, researchers could clearly see how wild boar behaved throughout the seasons. The animals showed different feeding habits at different times of the year, such as eating grasses in spring, cereals in summer, and root crops in late autumn. Three main patterns of damage were found. In spring, damage was rare but often very serious. In late summer and autumn, it happened more often but was usually less harmful. In some periods, like winter or late spring and early summer, boar were less active in fields. Knowing when and where wild boar are most active could help farmers and wildlife managers reduce both financial losses from crop damage and health threats to livestock from diseases like African Swine Fever.

## 1. Introduction

There are increasing reports of conflicts between humans and wildlife around the world, encompassing a wide range of interactions. These include attacks by predators on livestock and humans [1,2,3,4], transmission of diseases from wildlife populations to livestock [5,6,7], and road collisions [8,9]. Among these conflicts, one of the most widespread and impactful is crop damage caused by many species, leading to serious economic and social consequences. Such conflicts arise wherever wildlife habitats overlap with agricultural areas, regardless of agricultural intensity, human population density, or the level of urban development. This pattern is observed across different agricultural systems, from traditional and extensive farming in Africa [10,11] to large-scale monocultures in North America [12,13], densely populated agricultural landscapes in Asia [14,15], and technologically advanced farming in Europe [16]. Foraging in agricultural fields is a natural behavior of several wild animals, resulting from their optimal foraging strategy [17]. It is an outcome of evolutionary adaptations to changes in the availability of food resources [18,19]. As a result of anthropogenic landscape transformations, many wild animal species, especially herbivores, have adjusted their foraging habits to seasonal fluctuations in food availability, including resources derived from agricultural activities [20]. This often leads to conflict situations.

In Europe, many species, both mammals [21] and birds [22] cause damage to agricultural crops. Among them, wild boar (*Sus scrofa*) are particularly problematic, as they are among the most widespread and abundant wild ungulates worldwide. After centuries of persecution, the wild boar population in Europe expanded considerably in the second half of the 20th century, both in terms of numbers and range. This process is attributed to their exceptional ability to adapt to different environmental conditions, climate change, and reduced predation pressure, especially from wolves [21,23,24]. The population increase has also been enabled by shortcomings in the management of wild boar populations, such as inaccurate methods for estimating population growth and numbers and, in some cases, deliberate actions by hunters aimed at maintaining large populations of this attractive game species by means of supplementary feeding [25]. For many years, hunting plans did not take into account the high reproductive potential of wild boar, which can reach 250–300% of the spring population under favorable conditions [25,26]. As a result, the population grew uncontrollably. Despite extensive decimation measures in connection with African Swine Fever (ASF) at the beginning of the 21st century, wild boar are still the most numerous wild ungulate species in Europe [23,27,28].

As opportunistic omnivores, wild boar effectively adapt their diet to the availability of food [29,30]. Although they mainly inhabited forests in the past, they are increasingly invading non-natural ecosystems such as agricultural fields, meadows, and urban fringes due to their demographic increment and anthropogenic environmental changes [23,31,32]. The use of agricultural crops by wild boar has probably developed in parallel with the development of agriculture [33]. Agricultural landscapes, which cover almost 40% of Europe, provide wild boar with both a rich food source and a relatively undisturbed environment. This expansion and the intensive use of agricultural crops lead to significant yield losses, resulting in economic consequences and social conflicts [34,35,36,37].

Various methods are used to contain the damage caused by wild boar in agriculture, including physical barriers such as fences and electric enclosures [38], chemical repellents [39], light stimuli [40], and acoustic deterrents [41,42]. However, their effectiveness is often limited [19], and even protected crops remain susceptible to damage. The most commonly used strategy is therefore still the shooting of wild boar, an invasive method that is becoming increasingly socially unacceptable [43,44,45].

The intensity of the damage can vary depending on the year, season, and environment [46]. Understanding the basic temporal and spatial patterns of damage occurrence is crucial for developing effective strategies to manage wild boar populations and minimize their impact on agricultural landscapes. Systematic monitoring of damage and analysis of seasonal dynamics not only provides valuable data for wildlife management and agriculture but also serves as an important tool for the management of wider ecosystems. In particular, accurate knowledge of damage distribution can support efforts to control infectious diseases such as ASF, which poses a serious threat at the interface between wildlife and livestock.

The aim of this study was to provide detailed data on the seasonal damage dynamics and foraging preferences of wild boar in the agricultural landscape of central Poland, where this issue arises in ASF-affected areas. The study is based on one of the longest time series published to date, covering 20 years, during which damage was assessed using the same methodology, and the structure of crops in the agricultural landscape remained largely unchanged. This allowed a reliable assessment of the repeatability, regularity, and seasonal variation of damage in relation to different crop categories. In addition, an approach to wild boar population management was proposed that takes into account the need for population control, the reduction of damage in agricultural crops, and the increasing social disapproval of radical measures such as depopulation.

## 2. Materials and Methods

### 2.1. Study Area

**Figure 1:**
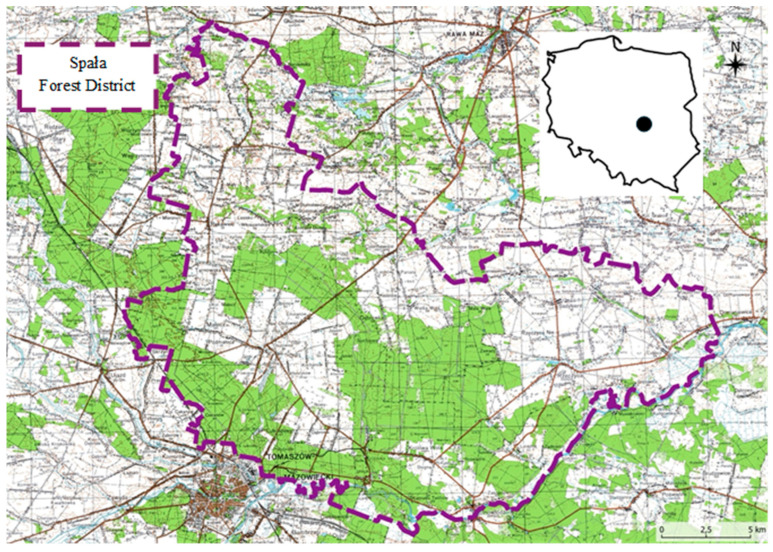
Location of forests (green) and agricultural lands (white) where wild boar damage was recorded from 1998 to 2020 in the Spała Forest District.

Data for research on the dynamics of damage caused by wild boar come from the hunting ground administered by the State Forest Service in the Spała Forest District (SFD) in central Poland (Figure 1).

The research area covers 15,585 hectares, of which 10,159 hectares is a dense forest complex and 5426 hectares is arable land. The forest stands of the SFD, shaped by centuries of human activity, are dominated by Scots pine (*Pinus sylvestris*), which covers over 70% of the area. However, in terms of forest sites, relative fertile habitats prevail, namely fresh mixed coniferous forests and fresh mixed deciduous forests. The characteristic plant communities include *Leucobryo-Pinetum* in coniferous and *Tilio-Carpinetum* in deciduous forest. A common feature of both is the locally well-developed shrub and ground vegetation layer, which provides favorable cover and food supply for large ungulates. According to annual spring estimates performed by State Forest Service officers, the estimated number of wild boar in the research area is approximately 400 individuals (2.5 individuals/km^2^). The annual hunting quota for wild boar averages 300 individuals (1.9 individuals/km^2^). Hunting in this area is conducted year-round. Battues are organized from October 1 to the end of January, while individual hunts take place throughout the year. During the summer, when agricultural fields suffer increased damage, wild boar are culled outside the forest to prevent further harm. In the autumn and winter, culling also occurs within the forest at bait stations. Hunters have approximately 60 high seats available for use. The agricultural areas surrounding the forest complex consist of a network of small farms, each averaging about 1 hectare. Agricultural crops are predominantly grains, which cover over 60% of the area. Due to intensive cattle breeding, meadows and pastures make up the second largest land use, approximately 22%. Potato crops occupy around 9% of the area, and corn, which is becoming increasingly popular, accounts for about 1% of the land. Legume crops, highly attractive to wild boar, also occupy a small portion of the area, approximately 2%. There are no major urban centers in the research area. However, the forests of the SFD attract many tourists, especially during the autumn mushroom-picking season, which is very popular in Poland.

### 2.2. Data Collection

The rules for reporting and estimating damage to agricultural crops in Poland are specified in the regulations of the Minister of the Environment [47,48], which is a normative act related to Polish hunting law [49]. According to these regulations, in hunting districts leased by hunting clubs, damage is assessed by hunters, who are also responsible for paying compensation to farmers. Upon the initiative of hunters or farmers, in the case of estimating the problematic damage (such as expensive crops, large area, registered presence of several ungulate species, etc.), independent appraisers—typically internal experts offering paid services—may be brought in, usually at the hunter’s expense. In the case of hunting districts managed by State Forest Districts, as was the case in SFD, specialized employees of the Forest District are responsible for assessing the damage, and compensation is paid by the Forest District. In SFD, throughout the entire research period, the same three properly trained individuals were responsible for this task.

The actual level of damage to agricultural crops may sometimes be underestimated because farmers cannot always pursue their claims. This occurs when farmers have not harvested their crops within 14 days from the end of the harvesting period for the specific plant species in each region, as determined by a resolution of the local government. Compensations are not paid to farmers who did not consent to the construction of devices (e.g., high seats) or the implementation of measures to prevent damage, or in the case of landowners who did not consent to hunting on their land. Compensations are also not paid when farmers store agricultural produce in stacks, mounds, or heaps in the immediate vicinity of the forest or when agricultural crops are not tended to in accordance with agricultural practices. However, such situations described above occur sporadically and do not significantly distort the quantitative and qualitative scale of damage in the SFD.

The damage registration procedure consists of three stages. Firstly, each farmer records the damage himself and reports it to the Forest District immediately after its occurrence. Forest District employees are then obligated to inspect the damage within 7 days of its reporting by the farmer. During the inspection, in addition to administrative matters, the type of crop, the perpetrator of the damage (determined based on tracks, feces, rooting, etc.), and the extent of damage are identified. The main perpetrator is determined based on the most numerous characteristics and fresh signs of animal presence. In cases where traces of different species (e.g., wild boar and red deer) are present, and the damage cannot be clearly attributed to a single species, the damage report records “wild boar/red deer”; in the case of orchard damage, for example, “moose/red deer”. In our study, we excluded from the analysis all cases (approximately 10% of damage involving wild boar) where the perpetrator was identified as “wild boar/red deer”. At this stage, measures are also taken to prevent further damage and protect crops. The protection methods most frequently used in Poland include electric fences, scent repellents, and concentrating hunting activities in each area. The third stage involves valuation, where the value of compensation is determined at the time of harvest by calculating the difference in yield obtained from the area damaged by game and the undamaged area in the same field.

Data on damage to agricultural crops in SFD were collected regularly for 21 years, from 1998 to 2020. However, in 2010 and 2011, technical modernization prevented these data from being collected electronically, and settlements for damage were carried out directly with farmers. Therefore, these data were not included in the analyses.

### 2.3. Data Elaboration and Analysis

The collected material allowed for the characterization of damage caused by wild boar to agricultural crops. This was further organized into 4 categories covering crops similar in terms of taxonomy or type. These categories were PASTURES, GRAINS, LEGUMES, and ROOT CROPS. A few rare cases of crops infrequently damaged by wild boar were included in the OTHER category and omitted from further analyses. The following were analyzed: (1) quantitative dynamics—changes in the number of registered cases of damage; (2) area dynamics—considering the areas damaged by wild boar; and (3) qualitative dynamics, referred to as “severity of damage”, which was the proportion of the total damaged area to the field area where the damage was registered. The severity of damage ranged from 0.01 to 1.00, indicating damage from 1% to 100% of the field surface. All values were presented as mean ± SD standard.

Temporal dynamics were analyzed in relation to monthly changes throughout the year and in longer time intervals, distinguished by comparing the number of registered cases of damage and the severity of damage. There were three patterns of damage: the first was termed *rare and severe* when the damage was relatively infrequent but severe; the second was termed *frequent and weak* when the damage was frequent but less severe; and the third, intermediate period, was termed *indirect*. It also analyzed how the composition of damage changes throughout the year in relation to various categories of crops and whether the field area affects the severity of the damage, and if so, in what time periods of the year and for which categories.

The Statistica ver. 13.3 package (StatSoft Polska™, Kraków, Poland) was used in statistical analyses. Spearman’s correlation was used to assess the relationship between crop area and severity of damage. When comparing means, the Median Test was used with the Kruskal–Wallis test as a post hoc test in the case of more than two variables and the Mann–Whitney U Test when comparing the means of two analyzed features. The significance of differences and the strength of relationships were determined at the level of *p* < 0.05.

## 3. Results

During 20 years of monitoring, 9871 reports of damage caused by wild boar were registered in SFD The area of agricultural fields where damage was recorded was relatively small, which results from the historical and demographic conditions of this part of Poland. The average field area was 1.04 ± 1.56 ha, and the field area distribution was strongly right-skewed with a dominant component of small-area fields (min = 0.01, Q25 = 0.28, Me = 0.56, Q75 = 1.12, max = 27.2). Damage from wild boar occurred in crops with a total area of 10,309.87 ha, while the area destroyed by wild boar in these fields amounted to 2343.17 ha. The average area of damage was 0.24 ± 0.43 ha. The smallest damage covered an area of 0.01 ha, while the largest area completely damaged by wild boar was 15.0 ha and took place in meadows. The average severity of damage was 0.34 ± 0.32, which means that, on average, approximately 34% of the crop area was destroyed by wild boar. The severity of damage to individual fields was, in most cases, very low or low (min = 0.01, Q25 = 0.11, Me = 0.20, Q75 = 0.49, max = 1.00). In 50% of cases (4935 records), the severity of damage did not exceed 0.20, i.e., 20% of the field area. In 448 cases (approx. 4.5% of all damage reports), all fields were destroyed by wild boar (Table 1). Damage caused by wild boar was recorded in 23 types of agricultural crops, which were divided into 5 categories: PASTURES—a category covering only grasses in meadows and pastures; GRAINS, including 9 species of cereals; LEGUMES, 4 species; ROOT CROPS, with 4 species of root-tuber crops and the OTHER category, which includes single crops of rare species such as rape, various vegetables, currant plantations, oil plants, and mustard. Crops from the OTHER category were the least frequently damaged by wild boar with 39 reports, but sometimes both the area of crops and the severity of their damage were very high. This was mainly due to rape, vegetables, and oilseeds, which they liked more than average.

### 3.1. Categories of Crops

Significant differences were found between the crop categories by comparing the means: crop areas (χ^2^ = 1616.58, *p* < 0.05), damage areas (χ^2^ = 151.69, *p* < 0.05), and severity of damage (χ^2^ = 847.76, *p* < 0.05) (Table 1).

In the case of average crop areas, the largest areas were occupied by rarely damaged crops from the OTHER category (2.39 ± 2.53 ha). They were comparable to the average areas of GRAINS (1.51 ± 1.90 ha) and LEGUMES (1.37 ± 1.55 ha) (*p* > 0.05). The average area of PASTURES (0.75 ± 1.25 ha) was significantly smaller than the three categories mentioned above (*p* < 0.05) but larger than the smallest areas of ROOT CROPS (0.45 ± 0.39 ha) (Kruskal–Wallis H = 1978.59, *p* < 0.05).

The largest areas of damage were also observed for crop plants from the OTHER (1.26 ± 1.64 ha) and LEGUMES (0.73 ± 1.10 ha) categories, the means of which did not differ significantly. However, they were larger than the damage recorded in the other three categories. Damage to GRAINS amounted to an average of 0.25 ± 0.40 ha and was statistically significantly larger than the average area of damage to PASTURES (0.22 ± 0.49 ha) and to ROOT CROPS (0.19 ± 0.49 ha) (Kruskal–Wallis H = 276.06, *p* < 0.05).

In terms of severity of damage, the greatest and similar levels of damage were found in three crop categories. On average, destruction covered approximately 50% of the field area: OTHER (0.63 ± 0.41), LEGUMES (0.55 ± 0.36), and ROOT CROPS (0.48 ± 0.36) (*p* > 0.05). The lowest severity of damage was recorded in the GRAINS (0.23 ± 0.25).

### 3.2. Crop Types

Differences in crop area and severity of damage were observed among 23 species of plants across the analyzed categories. The exception was the PASTURES category, which included only one crop type—meadows and pastures (Table 1).

In the GRAINS category, three dominant crop groups were distinguished. The most numerous and covering the largest areas were cereal mix (*n* = 1044, sum = 1693.52 ha), rye (*n* = 1036, sum = 1327.72 ha), and triticale (*n* = 973, sum = 1955.42 ha). Damage in these crops accounted for 67.9% of all reported cases in this category. The second group consisted of oat (*n* = 562, sum = 633.76 ha), barley (*n* = 421, sum = 692.15 ha), and wheat (*n* = 387, sum = 377.54 ha), contributing to 30.5% of damage reports. The least damage occurred in maize (*n* = 63, sum = 103.67 ha), buckwheat (*n* = 4, sum = 3.59 ha), and millet (*n* = 1, sum = 0.46 ha), together comprising only 1.6% of reports.

The variation in crop areas reflects the structure of crops in the study area, primarily resulting from the predominance of poor soils, which limit the cultivation of cereals to several main types. Differences in average field sizes among crops ranged from 0.4 ha for millet to 2.00 ± 2.62 ha for triticale. Larger average fields were also observed for maize (1.65 ± 2.27 ha), barley (1.65 ± 1.72 ha), and cereal mix (1.62 ± 2.03 ha), while smaller areas were associated with rye (1.28 ± 1.30 ha), oat (1.13 ± 1.23 ha), wheat (0.98 ± 1.12 ha), and buckwheat (0.90 ± 1.11 ha).

The severity of damage in the GRAINS category varied significantly among crops (Kruskal–Wallis test: H = 45.2, *p* < 0.001). The highest severity was noted for maize (0.54 ± 0.39), followed by wheat (0.33 ± 0.30). The lowest severity was observed for rye (0.21 ± 0.24) and barley (0.20 ± 0.20), with similar values for cereal mix (0.22 ± 0.21), triticale (0.23 ± 0.26), and oat (0.24 ± 0.21). Buckwheat (0.59 ± 0.33, *n* = 4) and millet (0.65, *n* = 1) exhibited high damage severity, but limited sample sizes prevent robust conclusions. Total destruction of crops occurred in varying proportions: cereal mix—3%, oat—3%, barley—3%, rye—6%, triticale—8%, wheat—11%, buckwheat—25%, and maize—30%.

The LEGUMES category included lupin, serradella, fodder peas, and other legumes. Damage was reported in lupin over a total area of 35 ha (*n* = 35) and in serradella across 18.70 ha (*n* = 31). Fodder peas were grown on 14.76 ha but sustained damage in only 6 cases. The remaining 58 reports pertained to fragmented crops classified as other legumes. The average area of fodder peas (2.46 ± 1.85 ha) and other legumes (1.96 ± 1.93 ha) was larger than that of lupin (0.9 ± 0.54 ha) and serradella (0.9 ± 0.76 ha).

Damage severity in the LEGUMES category showed limited variation but differed between crops (Kruskal–Wallis test: H = 23.8, *p* < 0.001). The lowest average severity was recorded for serradella (0.41 ± 0.31). The severity of lupin (0.66 ± 0.35) and other legumes (0.55 ± 0.35) was like fodder peas (0.72 ± 0.40), which experienced the most severe damage. Total crop destruction was most frequent for fodder peas (50%), followed by lupin (34%), other legumes (26%), and serradella (13%).

The ROOT CROPS category was dominated by potatoes (*n* = 2057, total = 927 ha), which accounted for most damage reports. Damage was also noted in carrots (*n* = 62, total = 22.4 ha), beets (*n* = 17, total = 4.64 ha), and two cases classified as other roots (*n* = 2, total = 1.29 ha). The average crop areas did not differ significantly and remained small (Kruskal–Wallis test: H = 5.1, *p* = 0.165): potatoes—0.45 ± 0.39 ha, carrots—0.36 ± 0.32 ha, and beets—0.27 ± 0.26 ha. All crops were cultivated for household purposes, as indicated by minimal field sizes (0.01 ha for potatoes, 0.02 ha for beets, and 0.03 ha for carrots). Damage severity was similar across crops, ranging from 0.48 (potatoes and beets) to 0.50 (carrots) and 0.61 (other roots) (Kruskal–Wallis test: H = 7.8, *p* = 0.049). Total crop destruction occurred in 23% of potato fields, 13% of carrot fields, and 18% of beet fields.

The OTHER category included rapeseed (*n* = 16, total = 45.25 ha), vegetables (*n* = 13, total = 21.40 ha), currants (*n* = 5, total = 13.00 ha), oil plants (*n* = 3, total = 12.30 ha), and mustard (*n* = 2, total = 1.09 ha). Apart from mustard, these crops were cultivated for trade and covered relatively large areas. The average cultivated areas were largest for oil plants (4.10 ± 4.55 ha), followed by rapeseed (2.83 ± 2.63 ha), currants (2.60 ± 2.16 ha), and vegetables (1.65 ± 2.09 ha). Damage to these crops occurred sporadically and was excluded from further analysis.

**Table 1:** Characteristics of 9871 crops damaged by wild boar in 1998–2020 in Spała Forest District.

Crop				Area of Crops [ha]					Damaged Area [ha]					Severity of Damage [Damage/Area]			
Category	*n*	Type	*n*	Mean	±SD	Sum	Min.	Max.	Mean	±SD	Sum	Min.	Max.	Mean	±SD	Min.	Max.
PASTURES	3073	grass	3073	0.75	1.25	2294.84	0.01	24.00	0.22	0.49	674.37	0.01	15.00	0.40	0.33	0.01	1.00
GRAINS	4491	cereals mix	1044	1.62	2.03	1693.52	0.01	18.00	0.27	0.40	278.01	0.01	6.00	0.22	0.21	0.01	1.00
		rye	1036	1.28	1.30	1327.72	0.01	12.50	0.17	0.22	180.12	0.01	2.37	0.20	0.24	0.01	1.00
		triticale	973	2.01	2.63	1955.42	0.01	27.20	0.29	0.40	279.95	0.01	5.80	0.23	0.26	0.01	1.00
		oat	562	1.13	1.23	633.76	0.03	11.00	0.26	0.63	143.91	0.01	11.00	0.24	0.21	0.01	1.00
		barley	421	1.64	1.72	692.15	0.01	18.00	0.25	0.31	106.50	0.01	2.55	0.20	0.20	0.01	1.00
		wheat	387	0.98	1.18	377.54	0.02	10.00	0.25	0.30	95.47	0.01	2.50	0.33	0.30	0.01	1.00
		maize	63	1.65	2.27	103.67	0.01	12.48	0.57	0.70	36.10	0.01	3.00	0.54	0.39	0.01	1.00
		buckweat	4	0.90	1.11	3.59	0.14	2.50	0.29	0.20	1.14	0.10	0.50	0.59	0.33	0.20	1.00
		millet	1	0.46	-	0.46	0.46		0.30	-	0.30	0.30		0.65	-	0.65	0.65
LEGUMES	130	other legumes	58	1.96	1.93	113.56	0.16	10.20	0.99	1.44	57.46	0.10	9.49	0.55	0.35	0.05	1.00
		lupin	35	0.90	0.54	31.65	0.10	2.30	0.59	0.53	20.82	0.05	2.30	0.66	0.35	0.10	1.00
		serradella	31	0.60	0.76	18.70	0.08	3.70	0.29	0.68	9.01	0.02	3.70	0.41	0.31	0.10	1.00
		fodder peas	6	2.46	1.85	14.76	0.82	6.00	1.25	0.71	7.51	0.50	2.16	0.72	0.40	0.08	1.00
ROOT CROPS	2138	potatoes	2057	0.45	0.39	927.17	0.01	4.30	0.19	0.25	390.64	0.01	2.18	0.48	0.36	0.01	1.00
		carrot	62	0.36	0.31	22.40	0.03	1.50	0.16	0.14	9.91	0.01	0.60	0.50	0.31	0.06	1.00
		beet	17	0.27	0.26	4.64	0.02	1.03	0.13	0.19	2.22	0.01	0.69	0.48	0.38	0.05	1.00
		other root	2	0.65	0.02	1.29	0.63	0.66	0.39	0.34	0.78	0.15	0.63	0.61	0.55	0.23	1.00
OTHER	39	rape	16	2.83	2.64	45.25	0.01	9.00	2.22	2.13	35.57	0.01	7.00	0.88	0.28	0.15	1.00
		vegetables	13	1.65	2.09	21.40	0.07	8.00	0.61	0.54	7.89	0.07	1.88	0.63	0.41	0.13	1.00
		currant	5	2.60	2.16	13.00	1.20	6.40	0.52	0.79	2.62	0.05	1.92	0.14	0.11	0.03	0.30
		oil plants	3	4.10	4.55	12.30	0.20	9.10	0.90	0.96	2.70	0.20	2.00	0.46	0.47	0.17	1.00
		mustard	2	0.55	0.08	1.09	0.49	0.60	0.08	0.05	0.17	0.05	0.12	0.15	0.07	0.10	0.20

A ranking of crops by severity of damage allows them to be divided into three categories: low severity (0.14–0.30): currant, mustard, barley, rye, cereal mix, triticale, oat; medium severity (0.31–0.61): wheat, pastures, serradella, oil plants, potatoes, beets, carrots, maize, other legumes, buckwheat; and high severity (0.61–0.88): other roots, other vegetables, millet, lupin, fodder peas, rapeseed.

### 3.3. Seasonal Dynamics of Damage

**Figure 2:**
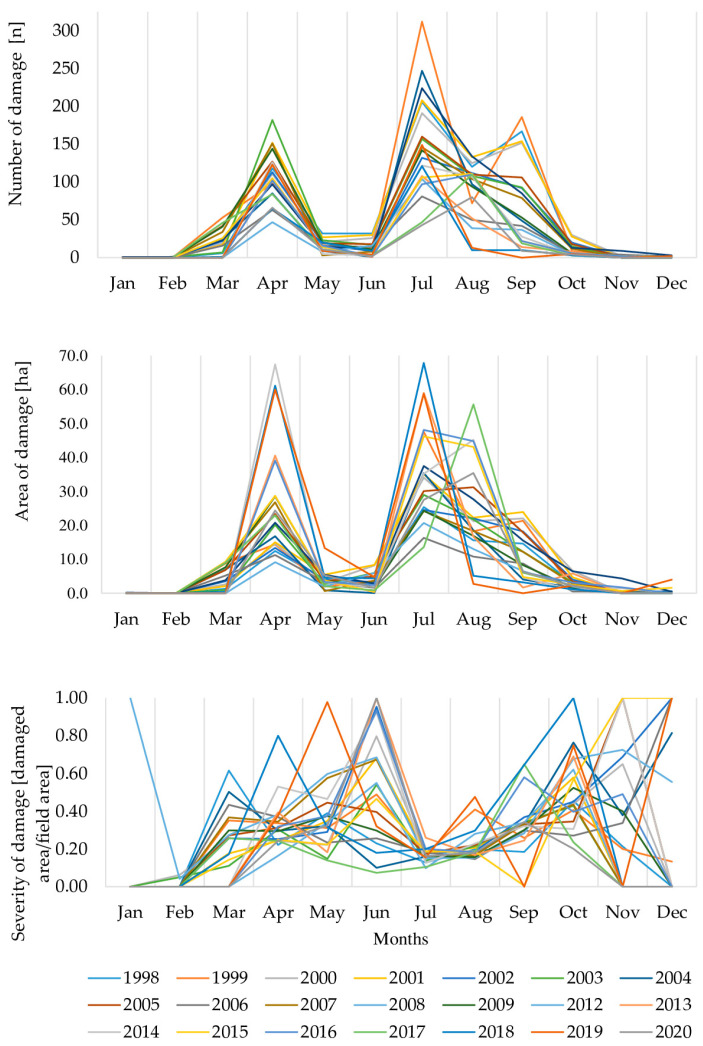
Yearly dynamics of numbers, areas, and severity of 9.871 cases of damage caused by wild boar in Spała Forest District in the years 1998–2020.

Three characteristics of damage—the number of reports, the area of damage, and the severity of damage—exhibited high monthly dynamics throughout the year (Figure 2).

The greatest variability occurred in the number of reports and the area of damaged crops, both showing two peaks. The first peak, in April, involved 2259 cases of damage (22.98% of all) over an area of 567.24 ha (27.7%). Following a calmer period in May and June, damage escalated again. The second peak, from July to September, included 6395 cases (65.0% of the total) affecting 1435.1 ha (62.6% of the total damage area). The months with the least damage were January (1 damage, 0.18 ha), February (3 cases, 0.11 ha), December (9 cases, 2.18 ha), and November (12 cases, 8.49 ha), followed by October (208 cases, 56.0 ha), June (227 cases, 67.81 ha), and May (328 cases of damage, 80.02 ha).

The severity of damage showed less pronounced dynamics compared to quantitative damage. Excluding January and February, when damage was sporadic, three patterns emerged. The first, frequent and weak, spanned July to September. During this period, the highest number of registered cases of damage (*n* = 6413) corresponded to the lowest severity: 0.23 ± 0.25, 0.29 ± 0.27, and 0.37 ± 0.31 for July, August, and September, respectively. Only 161 cases (2.3%) during this period involved complete crop destruction.

The second pattern, *rare and severe,* encompassed June and October to December (*n* = 458). Here, fewer cases of damage were recorded, but their severity was high, exceeding 50% of total crop damage on average. The mean severity for June, October, November, and December was 0.66 ± 0.39, 0.54 ± 0.35, 0.62 ± 0.33, and 0.72 ± 0.38, respectively. During this time, completely destroyed crops constituted 41.3% (*n* = 189) of all recorded cases of damage.

The third pattern, *indirect,* was observed in March, April, and May (*n* = 2969). These months showed moderate severity, but April’s cases of damage (0.42 ± 0.34)—were notably numerous, while March and May recorded fewer cases with severities of 0.42 ± 0.33 and 0.47 ± 0.33, respectively. In this pattern, 22.2% of the cases of damage involved complete crop destruction.

This division into three quantitative–qualitative damage patterns is a general classification and does not account for variations across specific crop categories. For example, most damage in pastures was *indirect*, while root crop damage was more frequently categorized as *rare and severe*.

The mean severity for *frequent and weak* damage, 0.28 ± 0.27, was significantly lower than for *indirect* damage, 0.43 ± 0.34 (Mann–Whitney U = 6,636,151, *p* < 0.05). The severity for *rare and severe* damage was the highest, 0.61 ± 0.38, and significantly exceeded both *frequent and weak* (Mann–Whitney U = 773,150.0, *p* < 0.05) and *indirect* (Mann–Whitney U = 54,431.0, *p* < 0.05).

**Figure 3:**
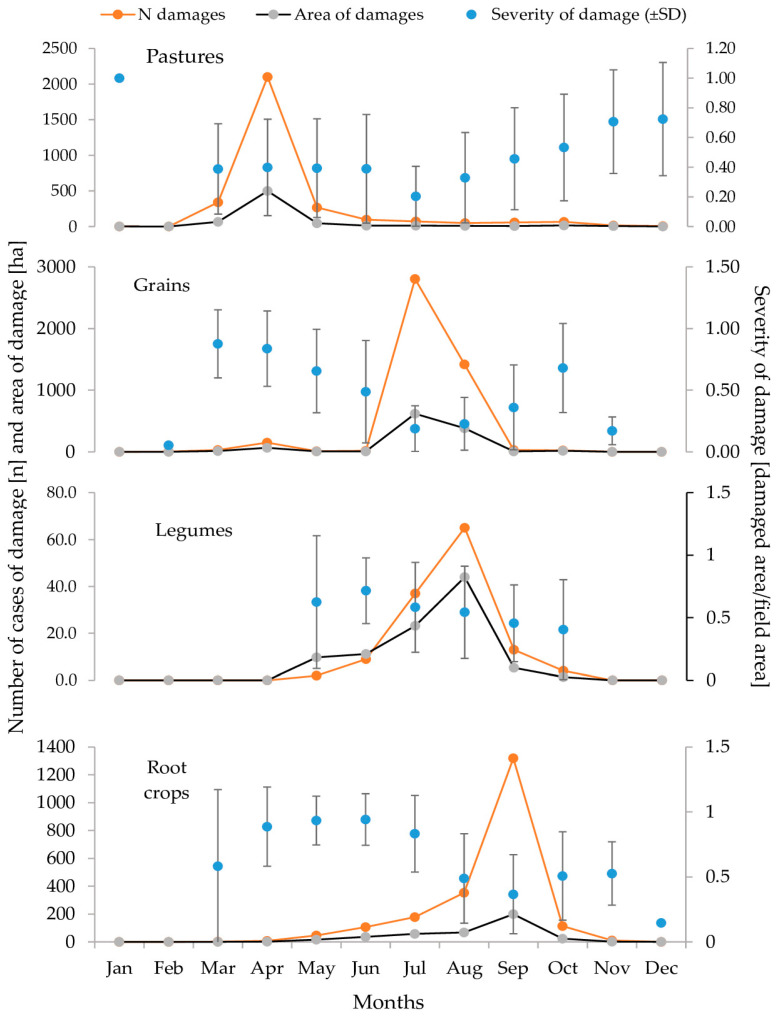
Changes in the number, area, and severity of damage to four crop categories over the year based on 9871 damage incidents recorded from 1998 to 2020 in Spała Forest District.

The monthly dynamics of damage were evident not only in total data but also across crop categories: pastures, grains, legumes, and root crops. Notably, the peak of the damage wave shifted over time and across crop types (Figure 3). This phenomenon was apparent in the number of reported cases of damage and damaged areas but not in the seasonal dynamics of damage severity.

Pastures experienced peak quantitative losses in April, with 2100 cases of damage covering 498.93 ha, representing 69.1% of all damage and 74.0% of the total damaged area for the year. Severity in April, 0.4 ± 0.32, was similar to March, 0.39 ± 0.30 (Mann–Whitney U = 355,281.0, *p* > 0.05), and May, 0.39 ± 0.33 (Mann–Whitney U = 276,453, *p* > 0.05). From May to December, severity increased, negatively correlated with the number of damage (r = −0.6, *p* < 0.05). By year-end, only 23 cases of damage (0.76% of all) occurred, yet severity reached 0.71 ± 0.35 in November and 0.72 ± 0.38 in December. Both values significantly exceeded April’s severity (Mann–Whitney U = 8123.5, *p* < 0.05 for April–November; Mann–Whitney U = 4197.0, *p* < 0.05 for April–December).

The second peak in damage intensity, from July to September, involved grains in July, legumes in August, and root crops in September. This sequence likely reflected wild boar preferences for crops at specific development stages (ripening or maturity) (Figure 3). In July, grains sustained 2804 cases of damage over 618.84 ha, 62.4% of all grain damage and 55.18% of the damaged area for the year. Winter crops experienced negligible damage from November to February, with only five cases recorded in the 20-year study period.

Severity trends in grains contrasted with quantitative dynamics. The most severe damage occurred in March, 0.88 ± 0.28, and April, 0.84 ± 0.31. Severity declined to a minimum in July, 0.19 ± 0.20 (Mann–Whitney U = 5299.50, *p* < 0.05 for July–March; Mann–Whitney U = 31,276.00, *p* < 0.05 for July–April). August maintained low severity at 0.23 ± 0.20 despite being the second-highest month for damage. After September’s harvest peak, the severity rose to 0.36. ± 0.34, significantly higher than in July (Mann–Whitney U = 29,580.00, *p* < 0.05). In October, only 23 cases of damage occurred, but their severity was high, 0.68 ± 0.36, as wild boar targeted freshly sown winter cereals.

Legumes showed a clear quantitative peak in August, with 65 cases (50% of all cases) over 94.8 ha (46.4% of the total damaged area). Severity remained stable, with no significant differences across months (χ^2^ = 7.519, df = 5, *p* > 0.05), though the damage was relatively severe. June recorded the highest severity, 0.72 ± 0.17, with an average of 72% destruction in affected crops.

Root crop damage peaked later, from August to October, coinciding with the maturity and harvest periods of potatoes. During these months, 1786 cases of damage (83.5% of annual cases) occurred over 290.14 ha (71.9% of the annual area). September saw 1319 cases of damage (61.7% of total) over 199.15 ha (49.3% of total). Severity in September, 0.37 ± 0.30, was lower than in August, 0.49 ± 0.34 (Mann–Whitney U = 184,166.0, *p* < 0.05), and October, 0.51 ± 0.34 (Mann–Whitney U = 55,367.0, *p* < 0.05). The highest severities occurred in April and May, 0.89 ± 0.19 and 0.93 ± 0.20, respectively, with fields often 90% destroyed.

### 3.4. Crop Composition

**Figure 4:**
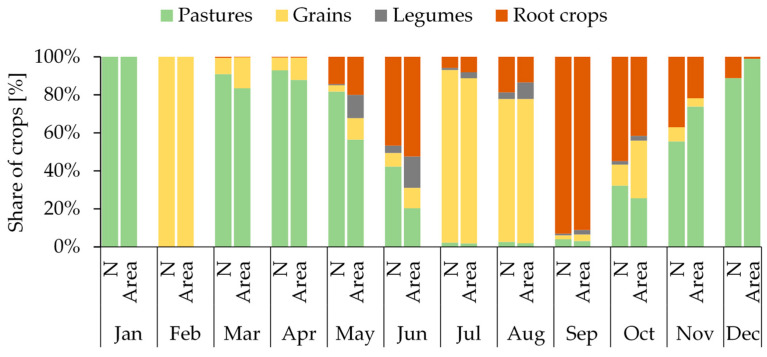
Structure of damage caused by wild boar to four categories of crops in subsequent months of the year in Spała Forest District during the years 1998–2020.

Large disparities were observed in the damage inflicted by wild boar across different crop categories in successive months of the year. The least variation in damage occurred in January and February, predominantly affecting PASTURES (100% damage in January) and GRAINS (100% damage in February). During spring, from March to May, when both the frequency and magnitude of damage notably increased (pattern: *indirect*), the damage was predominantly seen in pastures (*n* = 2682, constituting 90.6% of all cases in this period), with a much lower incidence in cereals (*n* = 183, 6.2%). Additionally, the first instances of damage to ROOT CROPS were recorded during this period (*n* = 57, 1.9%). In June (pattern: *rare and severe*), damage in PASTURES (*n* = 96, 42%) showed a clear decline. The first notable incident of wild boar attacks on ROOT CROPS was recorded (*n* = 106, 46.7%). There was also a notable proportion of damage in the LEGUMES category (*n* = 9, 4.0%), with minor damage reported in GRAINS (*n* = 16, 7.0%). June, akin to October, represented the peak period of diversity in terms of crop damage types. The *frequent and weak* period, spanning from July to September, was primarily characterized by the prevalence of damage in the GRAINS category (*n* = 4223, 66.0%) in July and August, followed by a surge in damage to ROOT CROPS post-harvest (*n* = 1319, 20.6%). Numerous damage was also recorded in LEGUMES during this timeframe (*n* = 115, 1.8%). The months of October, November, and December, constituting the latter part of the *rare and severe* damage pattern, exhibited a broad spectrum of crop damage. During this period, the interest of wild boar in PASTURES (*n* = 90, 36.9%) resurged, while damage to ROOT CROPS (*n* = 125, 51.2%) and GRAINS (*n* = 25, 10.2%) significantly decreased (Figure 4).

### 3.5. Area of Plantations and Severity of Damage

In most crop categories, weak but statistically significant negative correlations were observed between the severity of damage and the field area where damage was recorded. Wild boar typically caused more severe damage to smaller fields (up to 100%) compared to larger ones. The only exception was the relatively small but severely affected category of LEGUMES.

The average area of LEGUMES crops was 0.73 ± 1.10 ha, with an average damage severity of 55% (0.55 ± 0.36). The correlation between field size and damage severity was not statistically significant (r = −0.04, *p* > 0.05). This was consistent across all quantitative–qualitative damage patterns. During the first half of the year, when damage was rare but severe, 13 cases of LEGUMES damage were recorded, with an average field size of 1.76 ± 1.92 ha. The average damage severity was 62% (0.62 ± 0.33) and showed no significant correlation with field size (r = −0.07, *p* > 0.05). During periods of *frequent and weak* damage, 115 cases were recorded in LEGUMES, with an average field size of 1.26 ± 1.26 ha. The average severity was 55% (0.55 ± 0.36), again without a significant correlation between these variables (r = −0.06, *p* > 0.05). Damage during the *indirect* pattern occurred only twice, affecting large fields (average 5.62 ± 6.00 ha), which were neither representative of the category nor numerically sufficient to assess the relationship.

For PASTURES, the average field size was 0.76 ± 1.25 ha. The damage severity (mean 0.35 ± 0.33) was weakly, negatively, but statistically significantly correlated with field size (r = −0.39, *p* < 0.05). Field sizes did not differ significantly across damage patterns (χ^2^ = 3.97, df = 2, *p* > 0.05). *Rare and severe* spring damage occurred in fields averaging 0.66 ha (SD ± 1.06, *n* = 186), while *frequent and weak* summer–autumn damage affected fields averaging 0.86 ± 1.36 ha. *Indirect* damage, the most frequent (2709 cases), was recorded in fields averaging 0.75 ± 1.25 ha. Correlations between damage severity and field size were strongest for the *frequent and weak* pattern (r = −0.56, *p* < 0.05), followed by *rare and severe* (r = −0.49, *p* < 0.05), and weakest for *indirect* (r = −0.39, *p* < 0.05). Notably, 100% damage to PASTURES, primarily affecting the smallest fields, accounted for 11.9%, 17.2%, and 30.6% of *frequent and weak*, *indirect*, and *rare and severe* cases, respectively.

In the case of GRAINS, with an average field size of 1.51 ± 1.90 ha, a negative correlation was also observed between damage severity and field size (r = −0.39, *p* < 0.05). Field sizes were similar across damage patterns: 1.55 ± 1.89 ha for *frequent and weak*, 1.24 ± 1.31 ha for “*rare and severe*”, and 0.82 ± 2.02 ha for *indirect*, with a statistically significant difference noted for *indirect* (χ2 = 74.06, df = 2, *p* < 0.05). The strongest correlation was observed in 194 cases of *indirect* damage (r = −0.48, *p* < 0.05), followed by *rare and severe* (r = −0.35, *p* < 0.05, *n* = 41) and *frequent and weak* (r = −0.37, *p* < 0.05, *n* = 4253). The percentage of damaged fields varied: 2.6% for *frequent and weak* (fields up to 5 ha), 74.2% for *indirect* (fields up to 8 ha), and 31.7% for *rare and severe* damage (fields up to 2 ha).

**Figure 5:**
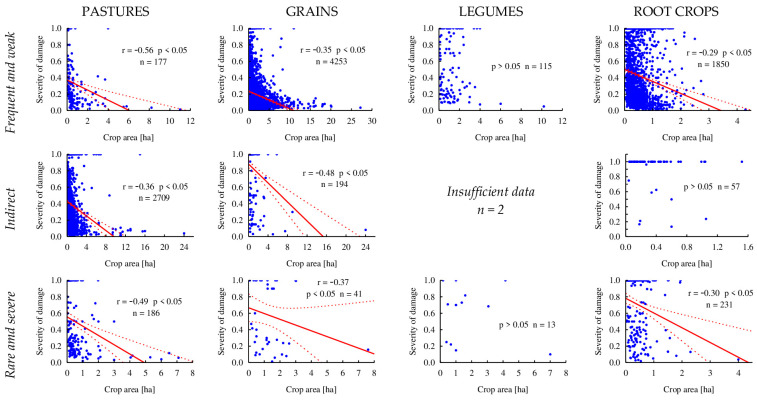
Correlations between the severity of damage and the area of fields in various crop categories across three quantity–quality patterns of damage caused by wild boar in Spała Forest District in the years 1998–2000 (dashed lines represent confidence intervals at *p* = 0.95).

For ROOT CROPS, with relatively small fields (mean 0.45 ± 0.39 ha), a weak but significant negative correlation was found between damage severity and field size (r = −0.29, *p* < 0.05). Field sizes across damage patterns were similar (χ^2^ = 4.95, df = 2, *p* > 0.05): 0.45 ha (±0.35) for *frequent and weak*, 0.36 ± 0.30 ha for *indirect*, and 0.42 ± 0.45 ha for *rare and severe*. Significant negative correlations were noted for *rare and severe* (r = −0.30, *p* < 0.05, *n* = 231) and *frequent and weak* (r = −0.29, *p* < 0.05, *n* = 1850), while the *indirect* pattern showed no significant relationship (r = −0.10, *p* > 0.05). The percentage of crops destroyed was 16.8%, 84.0%, and 51.5% for *frequent and weak*, *indirect*, and *rare and severe* damage patterns, respectively. Complete destruction predominantly affected the smallest fields (Figure 5).

## 4. Discussion

A study based on the analysis of 9871 wild boar damage records from the longest time series published to date—covering some 20 years of continuous records under virtually unchanged environmental conditions—allows a reliable assessment of the seasonal dynamics of this phenomenon and the species’ preferences for particular crops. In central Poland, wild boar damage showed high repeatability and a distinct bimodal pattern. The first peak occurred between March and the end of April, while the second occurred in the fall, in August and September, just before the harvest. In May, June, and partly in July, the number of cases of damage to agricultural crops decreased significantly. Agricultural fields thus served as a seasonal food source for wild boar, a pattern that has also been confirmed in other regions of Europe, including Luxembourg [35] or southern England [50]. The presence of two periods of increased damage supports previous findings that the diet composition of wild boar as opportunistic omnivores depends on three main factors: (a) energy requirements, (b) seasonality, and (c) the local availability of different food species [51,52,53]. Consequently, it was likely that forests and other non-agricultural areas provided food in lower quantity and quality in spring and during the ripening of cereal crops.

During the spring damage peak, wild boar in the SFD area foraged most intensively in the meadows and pastures for young grass stems, roots, and animals, including rodents and various developmental stages of insects [54]. Strong rooting of meadows was also observed in many other places in March and April [55,56,57,58]. This phenomenon can be attributed to several factors. One of them is the difference in the quantity and quality of food supply between meadows and other habitats accessible to wild boar, such as agricultural land and forests [59]. In forests emerging from winter dormancy, herbaceous vegetation developed later than in meadows, resulting in a lower food supply. In contrast, meadow and pasture soils were more biologically active in April: young, non-woody grass stems appeared in large quantities, while the roots continued to store nutrients, which could encourage wild boar to take root in these habitats [60,61,62]. Another important factor that favored the rooting of meadows in spring was soil moisture: at this time, the upper soil layers remained soft, which greatly facilitated rooting behavior [63].

It cannot be ruled out that the widespread feeding and baiting with maize—the crop most preferred by wild boar in Europe—in the SFD area was an additional factor that caused wild boar to forage intensively in the meadows in spring [34,51,52,62,64]. In hunting practice, maize is placed at baited sites throughout the year to increase the effectiveness of commercial hunting and reduce damage to agricultural crops by keeping wild boar in wooded areas. In SFD, this practice is applied to approximately 5000 hectares of forest in several dozen locations, where about 10 tons of corn are spread annually.

The correlation observed in Germany between the availability of maize and spring damage to meadows was explained by the fact that maize, due to its relatively low amino acid content, forces wild boar to seek animal protein—usually earthworms—to balance their diet [65]. A similar link was shown by Barrett (1978) [61], who argued that the search for earthworms in spring pastures could be due to the high carbohydrate content of maize. Considering that earthworms play an important role in the diet of wild boar [29,66,67,68], it can be assumed that spring pastures serve both as a reservoir of high-quality plant food (roots and aerial parts of grasses) and as an important source of amino acids in the form of animal feed. They can also be an important site for the extraction of water contained in plants and animal organisms.

The hypothesis linking the availability of maize in fields, its use as a feed supplement, and spring damage to meadows and pastures is partially based on studies of agricultural damage in Poland conducted since the 1970s. At that time, maize was not cultivated on the same scale as today, and potatoes were the dominant crop. Since their attractiveness to wild boar is comparable to that of maize, potatoes were then placed at bait sites year-round. The pattern of wild boar damage at that time showed a systematic increase—starting at a minimal level in spring and peaking between cereal ripening (July) and the end of the harvest season, including potato harvesting (September). Analyses of damage dynamics and characteristics in many regions of Poland indicated that even with high wild boar populations before maize cultivation became widespread, damage to meadows did not occur [69,70].

At the turn of May and June, the qualitative and quantitative conditions of the potential food supply for wild boar in meadows, fields, and forests changed. During this period, forests and other non-agricultural areas (e.g., wetlands and fallow land) offered a more diverse food supply than agricultural fields, where unripe cereals and pastures with increasingly fibrous grasses predominated. Sows rearing young during this period sought higher quality and more diverse food sources, leading them to rely more on baited sites and on herbaceous plants and grasses in woodlands and wetlands [54,71,72]. In addition, the activity of sows in caring for their young decreased, and these animals preferred areas with dense vegetation that provided them with cover [73]. Consequently, the intensity of crop damage in SFD decreased significantly from May to early July.

The situation changed again in July. The food resources available in the maturing fields began to exceed those available outside the fields. In the pre-harvest period, both sows with fast-growing offspring and other wild boar sought food in the cultivated fields, which at this time could yield several to several dozen tons of grain per hectare. Even in the richest forest ecosystems, these resources were practically unattainable, both quantitatively and qualitatively [27,74]. The increase in wild boar populations during this period—possibly two to three times higher than in spring—together with the availability of exceptionally abundant food resources, led to a second peak in damage intensity that reached the highest levels of the entire year. The seasonal dynamics of the damage were also accompanied by a clear variation in its severity.

Our study is the first to document three patterns of damage that differ both quantitatively and qualitatively: *rare and severe*, *indirect*, and *frequent and weak*. These patterns reflect the opportunistic foraging behavior of wild boar, which selects those that are most accessible at a given time depending on the availability and quality of food resources and compares them with alternative food sources [27,29,30,34,35].

The spring damage followed the pattern *rare and severe* and locally reached up to 80–100% of the affected area. They mainly occurred in meadows that provided high-quality forage for wild boar despite the limited overall food supply. Due to the small size of the affected area, wild boar foraging was highly concentrated during this period, resulting in significant losses.

In late summer, when food availability and diversity were at their highest, the *frequent and weak* pattern was observed. Although the number of losses was high, their severity averaged around 30%. In summer, the availability of food in agricultural crops is not only quantitatively high but also distributed over large areas, which limits the extent of damage at individual sites. Even with a two- to three-fold increase in wild boar populations compared to spring, the carrying capacity of agricultural ecosystems is so high that, despite the large number of cases of damage, each individual case remains relatively small. The overproduction of food in the cultivated fields during this period and the mild winters, which reduce natural wild boar mortality, further favor the population increase. In addition, the improved environmental conditions allow younger and younger females to successfully raise offspring, which can lead to an intensification of conflicts between wild boar and agriculture.

The *indirect* pattern characterized the periods between the damage peaks and was characterized by considerable fluctuations in both the severity and frequency of the damage. Both very heavy and minor damage was recorded during these periods, probably due to wild boar opportunistically encountering attractive crops rather than actively seeking them out and using them intensively in the absence of alternative food sources.

### Management Implications

The marked symmetry of seasonal damage dynamics and the preference of wild boar for different crops at different times of the year make it possible to propose a simple and practical method of population management. This method could help to regulate the density of wild boar and reduce damage to agriculture. To be effective and socially acceptable, it must take into account both the damage dynamics and the biology of wild boar. According to our findings, two periods have proven to be the most favorable for intensive hunting.

The first period, which favors effective hunting, is the damage peak in spring (*rare and severe*). During this time, wild boar prefer to stay in meadows, where they are relatively easy to find and hunt due to the lack of tall vegetation. In addition, wild boar populations reach their lowest annual numbers in March and April, making this a favorable time to regulate wild boar density before the breeding season and before the emergence of dense vegetation that would make them difficult to track down.

Another argument in favor of intensive spring hunting is that gestating females separate from last year’s piglets at this time, making it easier to distinguish between the two main age classes, piglets and older animals, including gestating females. The traditional practice of not shooting sows during the breeding season is considered an ethically and morally acceptable standard by Polish hunters, even though legal regulations allow the shooting of sows. The clear age structure of wild boar in spring allows a targeted focus on yearlings, which contributes to a reduction in population size and reproductive potential.

During the peak of the growing season in June and July (the *indirect* damage pattern), the wild boar population in agricultural fields increases sharply due to reproduction. Although this theoretically facilitates the detection of wild boar in the field, the high vegetation makes it difficult to detect piglets and to distinguish sows from other animals. For this reason, and for fear of orphaned piglets, hunters are generally reluctant to go hunting at this time. Furthermore, the damage is not yet serious enough to provide an incentive for increased hunting; instead, preventative measures such as fencing, repellents, and acoustic deterrents are generally used.

Another favorable period for intensive hunting is the second half of summer (the damage pattern *frequent and weak*) and winter. During the harvest period, when the plant cover decreases rapidly, it is easier to find wild boar in search of food. In addition, the piglets reach a sufficient size so that they can be tracked down more easily near the females, and if a sow is slaughtered, their chances of survival are much higher than in spring. In September, October, and November, hunting should aim to significantly reduce the wild boar population before the breeding season. Shooting should primarily target younger animals (yearlings) before the first rut. In late winter, piglets should also be included in the shooting measures.

The proposed solution is not only based on the need to limit agricultural damage. The recent history of ASF outbreaks in Eastern and Central Europe has highlighted significant shortcomings in the management of wild boar populations. The only measures that have been applied in practice to combat the growing wild boar population and the associated agricultural damage have been the lifting of hunting restrictions in most European countries prior to the outbreak of ASF and, following the appearance of disease foci, large-scale depopulation. Both approaches have caused considerable public controversy for understandable reasons. On the other hand, there is a lack of alternative, field-tested strategies for managing wild boar populations, which is likely to pose a new challenge for scientists and practitioners involved in wildlife management.

## 5. Conclusions

1. Analysis of 9871 cases over 21 years in a predominantly agricultural region in central Poland revealed a bimodal damage pattern. The first damage peak occurred in spring, mainly affecting meadows and pastures, while the second coincided with cereal ripening and harvesting in summer;
2. Three patterns of damage were identified: *rare and severe*—in spring, when food is limited; *frequent and weak*—in summer, with abundant food and cover; and *indirect*—diverse and rare damage in early spring and summer;
3. Seasonal feeding preferences of wild boar include grasses in spring, cereals in summer, legumes in early autumn, and root crops in late autumn;
4. Field size showed no significant effect on damage severity. Damage was typically less severe in larger fields, likely due to better food dispersion and reduced accessibility for wild boar;
5. To balance ethical concerns with effective population management, a dual-phase culling strategy is proposed. During spring (March–April), culling efforts should focus on yearlings, avoiding pregnant sows by targeting solitary animals, which are easier to identify during this period. In autumn (September–November), population density can be reduced ahead of the breeding season by targeting yearlings and late-born piglets.

Flexible and adaptive management strategies should align with wild boar feeding habits and damage patterns. Continued monitoring of agricultural and ecological changes is essential to refine and adjust population control methods effectively. 

## Data Availability

The data that support the findings of this study are available on request from the corresponding author.
